# Regional citrate anticoagulation (RCA) in critically ill patients undergoing renal replacement therapy (RRT): expert opinion from the SIAARTI-SIN joint commission

**DOI:** 10.1186/s44158-023-00091-w

**Published:** 2023-03-31

**Authors:** Valentina Pistolesi, Santo Morabito, Vincenzo Pota, Fabrizio Valente, Francesca Di Mario, Enrico Fiaccadori, Giacomo Grasselli, Nicola Brienza, Vincenzo Cantaluppi, Silvia De Rosa, Vito Fanelli, Marco Fiorentino, Marita Marengo, Stefano Romagnoli, the SIAARTI-SIN joint commission

**Affiliations:** 1 UOSD Dialisi, Azienda Ospedaliero-Universitaria Policlinico Umberto I, “Sapienza” Università̀ di Roma, Rome, Italy; 2 Department of Women, Child, General and Specialistic Surgery, University of Campania “L. Vanvitelli”, Naples, Italy; 3 Nephrology and Dialysis Unit, Santa Chiara Regional Hospital, APSS, Trento, Italy; 4 UO Nefrologia, Azienda Ospedaliero-Universitaria Parma, Dipartimento di Medicina e Chirurgia, Università di Parma, Parma, Italy; 5 Scuola di Specializzazione in Nefrologia, Dipartimento di Medicina e Chirurgia, Università di Parma, Parma, Italy; 6 Department of Anesthesia, Intensive Care and Emergency, Fondazione IRCCS Ca’ Granda Ospedale Maggiore Policlinico, Milan, Italy; 7 Department of Pathophysiology and Transplantation, University of Milan, Milan, Italy; 8 Department of Interdisciplinary Medicine, ICU Section, University of Bari “Aldo Moro”, Bari, Italy; 9 Nephrology and Kidney Transplantation Unit, Department of Translational Medicine (DIMET), University of Piemonte Orientale (UPO), AOU “Maggiore Della Carità”, Novara, Italy; 10 Centre for Medical Sciences-CISMed, University of Trento, Trento, Italy; 11 Anesthesia and Intensive Care, Santa Chiara Regional Hospital, APSS, Trento, Italy; 12 Department of Surgical Sciences, University of Turin, Turin, Italy; 13 Department of Anesthesia, Critical Care and Emergency, Città della Salute e della Scienza Hospital, University of Turin, Turin, Italy; 14 Nephrology Dialysis and Transplantation Unit, Department of Precision and Regenerative Medicine and Ionian Area (DiMePRe-J), University of Bari Aldo Moro, Bari, Italy; 15 Department of Medical Specialist, Nephrology and Dialysis Unit, ASL CN1, Cuneo, Italy; 16 Section of Anesthesiology and Intensive Care, Department of Health Sciences, University of Florence, Florence, Italy; 17 Department of Anesthesia and Intensive Care, AOU Careggi, Florence, Italy

**Keywords:** AKI, Citrate, CRRT, PIRRT, Regional citrate anticoagulation

## Abstract

Renal replacement therapies (RRT) are essential to support critically ill patients with severe acute kidney injury (AKI), providing control of solutes, fluid balance and acid–base status. To maintain the patency of the extracorporeal circuit, minimizing downtime periods and blood losses due to filter clotting, an effective anticoagulation strategy is required.

Regional citrate anticoagulation (RCA) has been introduced in clinical practice for continuous RRT (CRRT) in the early 1990s and has had a progressively wider acceptance in parallel to the development of simplified systems and safe protocols. Main guidelines on AKI support the use of RCA as the first line anticoagulation strategy during CRRT in patients without contraindications to citrate and regardless of the patient’s bleeding risk.

Experts from the SIAARTI-SIN joint commission have prepared this position statement which discusses the use of RCA in different RRT modalities also in combination with other extracorporeal organ support systems. Furthermore, advise is provided on potential limitations to the use of RCA in high-risk patients with particular attention to the need for a rigorous monitoring in complex clinical settings. Finally, the main findings about the prospective of optimization of RRT solutions aimed at preventing electrolyte derangements during RCA are discussed in detail.

## Section 1. Choice of anticoagulation modality for renal replacement therapies (RRT)

**Table:**
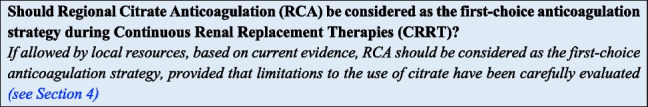

### Rationale

Critically ill patients admitted to the intensive care unit (ICU) are at high risk to develop acute kidney injury (AKI). The AKI-Epidemiologic Prospective Investigation (AKI-EPI) study reported an overall AKI incidence of roughly 57%; increasing AKI severity was associated with a higher mortality rate [1]. Furthermore, the AKI-EPI study showed an increased incidence of patients with severe AKI requiring renal replacement therapies (RRT) when compared to the previously published BEST Kidney international study (13.5% vs 4.3%) [1, 2]. RRT are essential to support critically ill patients with severe impairment of renal function, providing control of solutes, fluid balance and acid–base status [3, 4]. The different modalities of available RRT in this clinical context are continuous renal replacement therapies (CRRT), prolonged intermittent renal replacement therapies (PIRRT), or intermittent renal replacement therapies (IRRT), that are adopted depending on the clinical status, local expertise, and financial resources [4–9].

In most of the cases, an effective and prolonged anticoagulation during RRT is necessary to maintain the patency of the extracorporeal circuit [10]. Indeed, although RRT can be performed without anticoagulation, frequent circuit clotting is associated with blood loss, discrepancy between prescribed and delivered dialysis dose, increased workload, and costs [11, 12]. In terms of safety, anticoagulation should ensure a low risk of hemorrhagic complications, especially in critically ill patients where recent surgery, trauma and coagulopathy are common findings [13, 14]. Furthermore, AKI per se is associated with a higher bleeding risk [15].

Different strategies to prevent circuit clotting are applied, being systemic anticoagulation with unfractionated heparin (UFH) still the most widely used worldwide [5, 13]. However, several drawbacks may be associated with systemic anticoagulation, such as bleeding complications, heparin resistance and development of heparin-induced thrombocytopenia (HIT) [16–18].

Alternative anticoagulants for RRTs include the protease inhibitor nafamostat, not available in Europe [19, 20]. Nafamostat had been safely used, mainly in Japan and Korea, in the critically ill patients with increased bleeding risk [19, 20]. However, due to the absence of antidotes and its potential side-effects (e.g., agranulocytosis, anaphylaxis, hyperkalemia) nafamostat is actually not recommended for CRRT anticoagulation [21]. In order to limit the bleeding risk related to systemic anticoagulation, regional heparinization has been used in the past by combining prefilter heparin infusion with postfilter heparin neutralization with protamine [22]. However, this approach is cumbersome due to the difficulties of titrating heparin and protamine infusion rates; moreover, it exposes the patient to the side-effects of both drugs and can now be considered outdated [21].

Regional citrate anticoagulation (RCA), introduced in clinical practice for CRRT in the early 1990s [23], is an alternative strategy based on a fully loco-regional circuit anticoagulation. Since then, RCA has had a progressive diffusion and acceptance in parallel to the development of simplified and safe protocols [13, 24]. However, limited data are available to date about the real worldwide diffusion of RCA; indeed, the reported use of RCA ranging from 10 to 25% of CRRT in ICU derives from studies published more than 10 years ago [2, 5] or limited to a single country [25].

During RCA, citrate is infused at the beginning of the extracorporeal circuit (via the pre-dilution line) and acts by chelating the ionized calcium (iCa) in the blood, thus inhibiting the coagulation cascade. A significant amount of citrate-calcium complexes is then removed by diffusion and/or convection depending on CRRT modality and dialysis dose (30–60%). To avoid an excessive calcium loss leading to hypocalcaemia, calcium supplementation is needed [13, 24, 26].

The Kidney Disease Improving Global Outcomes (KDIGO) guidelines for AKI [21], based on few small randomized clinical trials (RCTs) [27–32], suggested to use RCA as the first line anticoagulation strategy during CRRT in patients without contraindications to citrate and independently from patient’s bleeding risk [21]. These suggestions have been endorsed by the Canadian Society of Nephrology and by a French multidisciplinary expert panel [33, 34], and more recently confirmed by a KDIGO Conference on controversies in AKI [35].

Due to the moderate or low-grade quality of the evidence supporting the 2012 KDIGO suggestions, new RCTs and meta-analyses have been published to investigate the efficacy and safety of RCA [36–40]. In 2016, a meta-analysis summarized the data from 14 RCTs, including 1134 adult patients with AKI requiring CRRT. The study showed a significantly prolonged filter lifespan in the citrate group and confirmed the superiority of RCA in reducing the bleeding risk. However, no difference in mortality was observed between the two groups (RR 0.97, 95% CI 0.84–1.13, *p* = 0.72) [41]. Similar results were reported in a recent meta-analysis that included 1229 patients from 16 RCTs [42].

In a recent multicenter RCT, 596 critically ill patients admitted to ICU and requiring CRRT were enrolled to compare the efficacy and safety of RCA versus systemic heparin anticoagulation. The RCA arm showed a prolonged filter lifespan (median 47 vs 26 h; difference 15 h, 95% CI 11 to 20, *p* < 0.001) and lower rates of bleeding complications (5.1% vs 16.9%; difference − 11.8%, 95% CI − 16.8% to − 6.8%, *p* < 0.001). No difference in 90-day all-cause mortality was reported between the 2 groups (Kaplan–Meier estimator percentages, 51.2% in the RCA group vs 53.6% in the heparin group, *p* = 0.38). However, no definitive conclusion could be drawn concerning mortality because the trial was prematurely terminated and for this reason underpowered [43]. These findings on the efficacy and safety of RCA further support the KDIGO suggestions and a wider diffusion of RCA. However, it should be considered that local availability (e.g., RCA not approved for CRRT use in USA) and potential contraindications to citrate use (see “Section 4. Potential limitations to RCA and monitoring in high-risk patients” section) may represent the real limitations to a more extended use of RCA. Furthermore, issues related to the costs of CRRT solutions dedicated to RCA could represent a potential concern for a more extensive use of this anticoagulation strategy. However, the lower bleeding complication rates and the longer filter lifespan could potentially lead to a global cost saving [44–46]. Moreover, local expertise and the wider use of semi-automated systems could allow to decrease the need for frequent monitoring, further limiting costs and nurse workload [46].

## Section 2. Renal replacement therapy modalities and the use of regional citrate anticoagulation

**Table:**
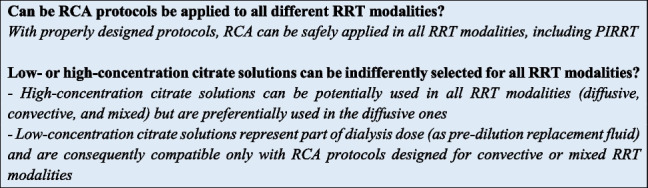

### Rationale

**Fig. 1:**
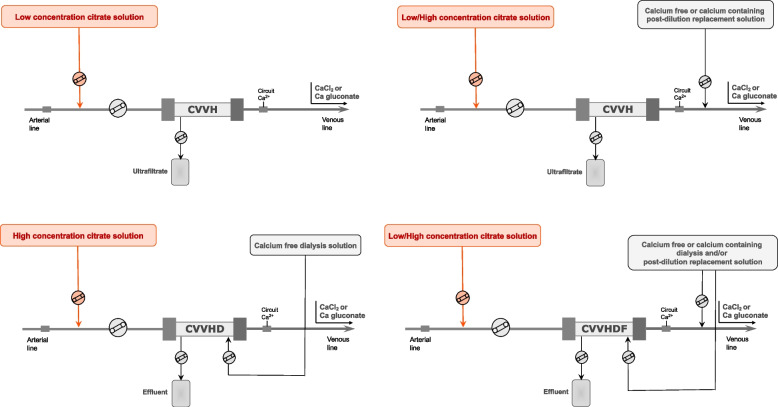
Simplified CRRT circuits with RCA for CVVH, CVVHD, and CVVHDF. Abbreviations: CRRT, continuous renal replacement therapy; RCA, regional citrate anticoagulation; CVVH, continuous venovenous hemofiltration; CVVHD, continuous venovenous hemodialysis; CVVHDF, continuous venovenous hemodiafiltration; Qb, blood flow rate

Due to their better hemodynamic stability, efficient fluid removal and adequate metabolic control, CRRT and PIRRT represent the primary choices in critically ill patients with AKI [47, 48]. Several modalities of RRT are available, each primarily depending on the mechanisms used for solute transport (i.e., diffusion, convection), which can act separately or in combination (Fig. 1) [3]. Continuous veno-venous hemodialysis (CVVHD) is a continuous modality exclusively based on diffusion; solute removal is mainly dependent on molecular weight, being smaller molecules more easily removed than larger molecules. Continuous veno-venous hemofiltration (CVVH) is the RRT modality that only relies on convection. Convection generates large ultrafiltrate volumes across the hemofilter by applying transmembrane pressure gradients, moving predetermined fluid volumes and solutes with them (solvent drag). In this case, both small and medium-size solutes are effectively removed. The contemporary adoption of both solute removal principles is characteristic of continuous veno-venous hemodiafiltration (CVVHDF), a third RRT modality that typically requires both dialysis and replacement fluids. Other modalities of RRT, commonly named PIRRT or sustained low efficiency dialysis (SLED), are characterized by sessions lasting 8–12 h, and share most of the advantages of both conventional IRRT and CRRT [48–50]. PIRRT, predominately based on diffusive mechanism for solute transport, has been shown to be safe and convenient, providing excellent control of electrolytes and fluid balance [50]. RCA can be easily used in all the above RRT modalities, albeit with different circuit setting depending on the citrate solution available [51].

**Fig. 2:**
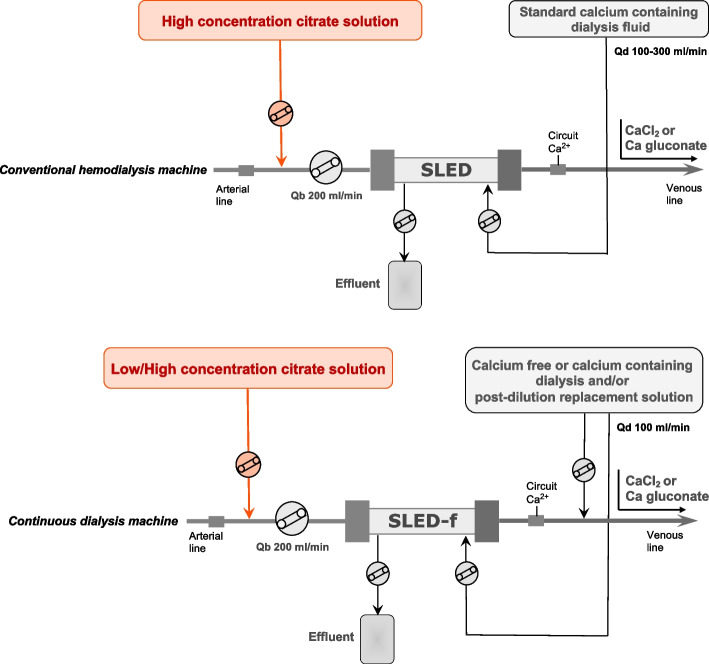
Simplified PIRRT circuits with RCA for SLED and SLED-f, variably applied with any conventional hemodialysis or CRRT machine. Abbreviations: PIRRT, prolonged intermittent renal replacement therapy; RCA, regional citrate anticoagulation; SLED, sustained low efficiency dialysis; SLED-f, sustained low efficiency dialysis filtration; Qb, blood flow rate; Qd, dialysis flow rate

Based on their citrate content, commercially available RCA solutions can be classified in high (hypertonic in sodium) vs. low-(isotonic in sodium) citrate concentration (Table 1) [26]. High citrate concentration solutions are generally adopted for diffusive modalities (CVVHD and SLED), whereas low-concentration citrate solutions are widely used in convective or mixed modalities (CVVH, CVVHDF) and in some SLED variants (e.g., SLED-f) (Figs. 1 and 2). To avoid the risk of hypernatremia and metabolic alkalosis, the hypertonic citrate solutions can be combined with customized dialysis solutions characterized by low sodium and low bicarbonate concentration aimed at optimizing electrolyte and buffer balance in CRRT [52]. During convective modalities, where protocols with isotonic citrate solutions are generally adopted, a high flow rate of the citrate-based fluid is needed to achieve the target levels in the extracorporeal circuit; hence, citrate-buffered replacement solution will significantly contribute to the total dialysis dose. The physiologic sodium content of isotonic solutions allows the use of standard sodium concentration replacement fluid and/or dialysate. The buffer supply derives from citrate (pre-dilution CVVH) or citrate and bicarbonate in various proportions (pre-dilution and post-dilution CVVH and CVVHDF) in relation to parameter settings and fluid combination [26]. The use of low concentration citrate in pre-dilution CVVH modality allows the prescription of CRRT by using only one solution and the use of RCA without a dedicated infusion pump. However, with this approach the dialysis dose is strictly related to citrate dose, possibly complicating the acid–base optimization, and increasing the risk of citrate accumulation when high dialysis dose is delivered [53]. Therefore, a common strategy to overcome these risks is to combine the isotonic citrate solution with post-dilution replacement fluids (CVVH) or both post-dilution replacement fluids and dialysis fluid (CVVHDF). Several RCA protocols, characterized by the combination of solutions with different buffer and electrolyte composition aimed at tailoring RRT prescription, have been published in the last decade [26, 54–57] (Fig. 1).

**Table 1:** Composition of main, commercially available, citrate solutions for RRT

Composition	Citrate solutions	ACD-A (different manufacturers)	Sodium citrate 4% (Fresenius Medical Care)	Citrasol 4%® (Braun)	Regiocit® (Baxter)	Citrachoice 24® (Medtronic)
Trisodium citrate (mmol/l)		74.8	136	136.4	18	20
Citric acid (mmol/l)		38.1	–	0.3 g/l		4
Sodium (mmol/l)		224	408	404.6	140	158
Chloride (mmol/l)		–	–	–	86	86
Glucose (g/l)		24.5	–	–	–	–

*RRT* renal replacement therapies availability and trade name of each solution may vary according to different countries

The application of RCA to PIRRT has been recently increasing, using either standard dialysis machines or CRRT monitors [51, 58]. By using a classical CRRT machine, PIRRT could be performed in diffusive or mixed modality by setting dialysate or effluent flow rate at 100 ml/min (Fig. 2).

Based on the peculiar setting of PIRRT, RCA can be applied safely also in patients at risk of citrate accumulation. Indeed, the potential citrate accumulation during the dialysis session is usually counterbalanced by the interdialytic phase in which citrate is not delivered, even in the presence of reduced liver function and decreased citrate clearance [51, 59]. Moreover, the high dialysate flow rate, which characterizes PIRRT, allows removing a relevant portion of citrate infused in the circuit (up to 70%) [59]. The most validated RCA protocols for SLED include the use of a high concentration citrate solution such as ACD-A combined either with calcium-free or calcium-containing dialysis solutions by using a conventional dialysis machine [59–62] (Fig. 2). In this regard, with the aim to avoid the risk of hypocalcemia and to simplify the handling of RCA protocol, the use of a calcium-containing dialysis fluid may significantly contribute to reduce the external calcium infusion trough calcium back-transport from the dialysis fluid [60].

## Section 3. RCA in specific extracorporeal organ support systems combined with RRT

**Table:**
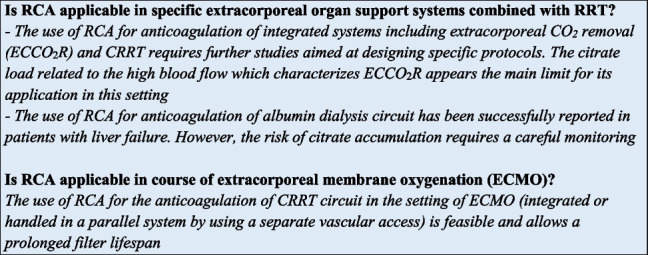

### Rationale

Several extracorporeal organ support systems, such as extracorporeal membrane oxygenation (ECMO), left ventricular assist device (LVAD), extracorporeal CO_2_ removal (ECCO_2_R), liver support systems and therapeutic apheresis could be used alone or in combination with RRT in specific ICU clinical settings [63]. The Extracorporeal Life Support Organization (ELSO) guidelines do not recommend a specific anticoagulant for ECMO, although systemic unfractionated heparin (UFH) is the most used; direct thrombin inhibitors (argatroban and bivalirudin) or an anticoagulation free protocol were considered only in the case of a contraindication to heparin [64]. For LVAD system, the current guidelines recommend the combination of anticoagulant therapy (warfarin) with antiplatelet therapy (aspirin) for the prevention of thrombosis and device failure [65]. ICU patients undergoing ECMO often develop severe AKI requiring RRT [66]. In the presence of two extracorporeal circuits (CRRT and ECMO), the contact of the blood with multiple non-biological surfaces may enhance the activation of the coagulation cascade; thus, an adequate anticoagulation strategy is required in this specific setting. As previously discussed, RCA is suggested as first choice option for CRRT circuits. However, RCA could not be conceptually applied as anticoagulation strategy for the ECMO circuit. Indeed, citrate flow rates required for the extremely high blood flow of ECMO (50–80 ml/kg/min)—much higher than the conventional range of 150–200 ml/min used in CRRT/PIRRT—would lead to systemic hypocalcemia and to a very high citrate load, largely overcoming physiological citrate metabolic rate. Although systemic anticoagulation represents the standard anticoagulation strategy during ECMO, RCA could be added in some cases to prevent repeated CRRT circuit clotting [67]. Indeed, RCA has been successfully used for anticoagulation of CRRT circuit in patients undergoing ECMO. In a prospective study conducted by Giani et al. the efficacy and safety of adding RCA to a CRRT circuit integrated in the ECMO system was compared to the use of the sole UFH [68]. The primary objectives of the study included filter lifespan, blood coagulation parameters and complications related to citrate. The authors showed that in the 22 patients treated with RCA + UFH the incidence of circuit coagulation was lower when compared with UFH group and there was no difference in terms of incidence of hypernatremia or outcome [68].

The current indication for the combination of CRRT and ECCO_2_R systems is the coexistence of respiratory acidosis and AKI. Considering the ECCO_2_R systems characteristics, the main issue for the use of RCA is linked to the citrate metabolic rate and to the blood flow rate necessary for an effective CO_2_ removal, which also in the latest generation systems is at least 0.4 L/min. With these systems, the role of citrate as an alternative to UFH is still undetermined and may be worthy of further investigation. In an animal model, local citrate anticoagulation was as effective as UFH but did not increase CO_2_ removal and led to increased incidence of hypocalcemia and acidosis [69].

Among liver support system, RCA has been proposed also in the setting of albumin dialysis [51]. However, considering the risk of citrate accumulation due to metabolism impairment, RCA use in liver failure patients undergoing liver support treatments is still controversial and needs further research [51]. Data available to date on patients undergoing albumin dialysis with RCA have shown its efficacy in terms of circuit lifespan and safety, provided that a careful monitoring is ensured [70, 71].

Finally, about the emerging adjuvant therapies for sepsis and septic shock, few studies have investigated the feasibility and safety of RCA to optimize the use of specific devices for these techniques (e.g., polymyxin-B hemoperfusion, oXiris membrane, cytokines hemoadsorption devices) [72–74]; due to the limited experience, further studies are needed to allow recommendation in this particular setting.

## Section 4. Potential limitations to RCA and monitoring in high-risk patients

**Table:**
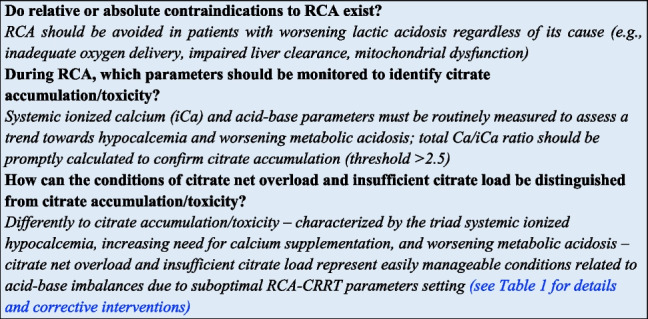

### Rationale

RCA is considered the first line anticoagulation strategy for CRRT in patients without contraindications [10, 75]. However, the implementation of RCA requires dedicated protocols and specific training of both medical and nursing staff.

Citrate accumulation is a feared and potentially lethal complication of RCA rarely occurring in the “average” critically ill patient with AKI, when a strict protocol is followed. Studies from centers with extensive experience with RCA report very low rates of RCA-related complications and citrate accumulation in unselected patients undergoing CRRT [76]. Although there are no absolute contraindications to RCA, this method should be applied with caution in patients with severe or worsening lactic acidosis (increasing trend in lactic acid serum levels) likely due to liver and systemic hypoperfusion and severe intracellular hypoxia (e.g., septic or cardiogenic shock), or to severe liver failure, including ischemia–reperfusion injury after liver transplantation.

**Fig. 3:**
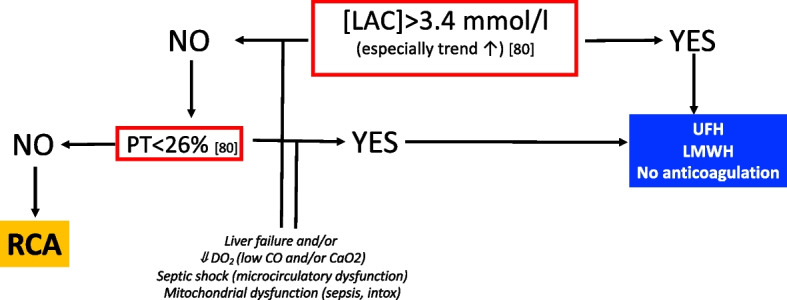
Proposed algorithm for the selection of anticoagulation modality during RRT. Abbreviations: LAC, lactate concentration; PT, prothrombin time; UFH, unfractioned heparin; LMWH, low molecular weight heparin; RCA, regional citrate anticoagulation

Hyperlactatemia might be the metabolic sign of severe liver failure, circulatory dysfunction and/or inadequate oxygen delivery (DO_2_) as in cardiogenic shock. Serum lactate > 3.4 mmol/L and/or need for high dose vasopressors (as markers of circulatory shock) have been indicated as factors to be carefully considered before starting RCA due to the risk of developing citrate intolerance [77–80]. Most importantly, the trend of lactate plasma concentration, rather than its absolute value, has been recently indicated as more informative (and predictive) about the risk of inadequate citrate metabolism [81] (Fig. 3).

Liver dysfunction has been historically considered a contraindication to the use of RCA due to deranged liver metabolism and the ensuing increased risk of citrate accumulation. Nevertheless, recent studies suggested that RCA may be safe even in patients with severe chronic liver insufficiency [82]. Some important clinical and technical aspects have led to reconsider liver failure as a contraindication for RCA: the role of extra-hepatic metabolic pathways (i.e. skeletal muscle and renal cortex), new software generations that help the clinicians in the management of RCA, and the modulation of convective and/or diffusive CRRT dose (increased removal of citrate with the effluent fluid).

Finally, some intoxications (e.g., biguanides like metformin, paracetamol, propofol, linezolid, tenofovir) can lead to mitochondrial dysfunction and transient decrease of citrate metabolism, as in part mentioned elsewhere [77, 83].

In high-risk patients and in clinical situations where citrate metabolism is markedly impaired, citrate-calcium complexes tend to accumulate, leading to metabolic acidosis and reducing plasma iCa concentration. In this condition, the impaired metabolism of citrate-calcium complexes leads to the clinical triad which characterized citrate accumulation: systemic ionized hypocalcemia, increasing need for calcium supplementation, and worsening metabolic acidosis. Systemic ionized hypocalcemia, despite a progressive increase of calcium supplementation, can be easily explained by the lack of calcium release from the unmetabolized calcium-citrate complexes. Otherwise, the occurrence of worsening metabolic acidosis requires specific considerations related to the basic principles of RCA. Indeed, RCA protocols are designed to optimize buffer supply throughout a circuit mass balance of citrate and bicarbonate that takes into account the indirect generation of bicarbonate from citrate metabolism. In case of citrate accumulation, the lack of indirect generation of bicarbonate from citrate invariably leads to a negative buffer balance and worsening metabolic acidosis due to an insufficient buffers supply.

Based on these considerations, the following parameters should be routinely monitored in all patients undergoing RCA-CRRT and even more frequently in high-risk patients. Post-filter and systemic ionized calcium should be measured to assess efficacy and safety, respectively, whereas total Ca/iCa ratio should be measured to promptly diagnose citrate accumulation (see below).

Recommended samples [77]:

- Circuit iCa (post-filter) every 6–8 h with a target of 0.25–0.40 mmol/l
- Patient serum iCa every 6–8 h with a target of 1.1–1.3 mmol/l
- Serum total Ca (and total Mg) every 12–24 h

Circuit iCa is the leading target to verify RCA efficacy whereas patient iCa should be measured in order to carefully set calcium compensation (replacement of calcium lost in the effluent bag).

In the absence of widely available measurement of blood citrate levels (the gold standard), the most reliable sign for citrate accumulation is an increased total/ionized calcium (Ca/iCa) ratio (> 2.5).

When an RCA strategy is applied, it is crucial for clinicians (both nurses and physicians) to detect citrate accumulation and distinguish it from other acid–base disturbances like citrate net overload and insufficient citrate delivery [82] (Table 2). If citrate administration exceeds the body’s capacity (liver, skeletal muscle, kidney cortex) to metabolize citrate (tricarboxylic acid cycle or Krebs’ cycle), progressive accumulation eventually occurs. As underlined above, signs of citrate accumulation include an increased effort aimed at maintaining physiologic levels of serum iCa concentration (i.e., increase in calcium substitution needs), worsening of metabolic acidosis, and, very important, a > 2.5 (or a trend towards this value) total/ionized calcium (Ca/iCa) ratio value. In the case of citrate accumulation, the risk for hypocalcemia must be carefully monitored, prevented, and eventually treated [26]*.*

**Table 2:** Differential diagnosis between citrate accumulation and other benign conditions

	Citrate accumulation	Citrate net overload	Insufficient citrate load
Origin	Reduced capacity to metabolize citrate	Excessive citrate administration/buffer needs	Insufficient citrate administration/buffer needs
Total Ca/iCa ratio	> 2.5	Normal (≤ 2.5)	Normal (≤ 2.5)
Metabolic acidosis/alkalosis	Acidosis	Alkalosis	Acidosis
CaCl2 administration	↑ (tendence to hypocalcemia)	Normal	Normal
Severity (risk)	High (hypocalcemia)	Low	Low
Frequency	Uncommon if excluding high-risk cases	Common	Uncommon
Complexity of correction	Complex	Easy	Easy
Possible interventions	↓ Q_B_ and/or ↑ Q_D_ and/or ↑ Q_R_^post^ ↓ target citrate dose (mmol_CIT_/L_B_) or RCA stopping with switch to alternative anticoagulation strategies	↓ Q_B_ and/or ↑ Q_D_ and/or ↑ Q_R_^post^ ↓ target citrate dose (mmol_CIT_/L_B_)	↑ Q_B_ and/or ↓ Q_D_ and/or ↓ Q_R_^post^ ↑ target citrate dose (mmol_CIT_/L_B_)

*Abbreviations*: Q_B,_ blood flow rate, *Q*_*D*_ dialysate flow rate, *Q*_*R*_^*POST*^ replacement fluid flow rate in post-dilution, *L*_*B*_ liters of blood flow rate, *iCa* ionized calcium, *CIT* citrate

Differently, net citrate overload is a benign and common condition that does not require drastic interventions, but rather adjustments of machine settings. Citrate overload is characterized by an integrity in the organism’s capacity to metabolize citrate that results in the development of metabolic alkalosis due to an increased production of bicarbonate from citrate anion metabolism in the Krebs’ cycle (citrate anion needs three hydrogenions to enter the cycle in the mitochondria, thus releasing three bicarbonate ions in the blood). Excessive citrate load and/or low clearance in the hemofilter can lead to net citrate overload [77].

In the case of inadequate citrate delivery, bicarbonate supply from citrate metabolism could be insufficient to adequately buffer the AKI-associated acidosis. Being this condition related to an imbalance between the citrate/bicarbonate delivered to the patient and the citrate/bicarbonate removed with the effluent, an appropriate modulation of main RCA parameters allows in most of the cases to tailor buffers balance according to clinical setting and patient needs [77]. Thus, a separate bicarbonate supplementation is generally not required to correct this acid–base imbalance. Importantly, Ca/iCa ratio is always ≤ 2.5 both in the case of net citrate overload and insufficient citrate delivery [77].

Noteworthy, medical and nursing education, ensuring an early recognition and management of high-risk patients, can prevent most accidental or unwanted cases of complications during RCA.

## Section 5. Optimization of RRT solutions to prevent electrolyte derangements during RCA

**Table:**
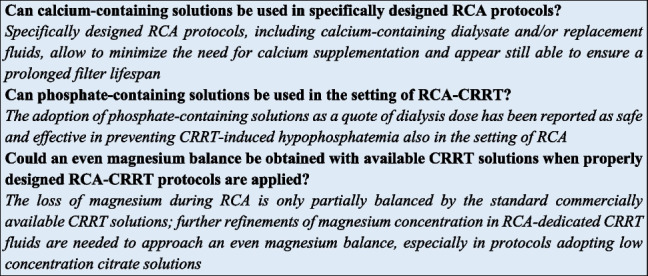

### Rationale

In the past, the use of off-label citrate solutions, not specifically dedicated to RRT, has limited a more widespread application of RCA for concern about the risk of electrolyte and acid–base disorders. However, the recent availability of commercial citrate solutions for CRRT has facilitated the use of RCA, also improving its safety in critically ill patients with AKI [26]. Indeed, a balanced combination of citrate and CRRT solutions, now available in different formulations, allows the modulation of buffer supply and electrolyte balance according to clinical needs, thus minimizing the risk of acid–base and electrolyte derangements (Table 3) [26].

**Table 3:** Optimization of main electrolyte balance during RCA-CRRT

Electrolyte	Potential derangement	Rationale	Optimization measures
Calcium	Hypocalcemia	RCA-RRT are characterized by a negative calcium mass balance requiring calcium supplementation	Calcium supplementation need during RCA-RRT can be significantly reduced by using calcium-containing dialysate and/or replacement fluid
Magnesium	Hypomagnesemia	RCA-RRT are characterized by a negative magnesium mass balance, especially with the use of low concentration citrate solutions	Magnesium supplementation should be properly ensured during RCA-RRT Magnesium concentration of CRRT solutions dedicated to RCA could be worthy of further optimization according to the protocol adopted
Phosphorus	Hypophosphatemia	Phosphate depletion is common in critically ill patients, especially during prolonged or continuous RRT modalities	Use of phosphate-containing dialysate and/or replacement fluids, as a variable proportion of dialysis dose, allows to minimize the need for phosphate supplementation

*RCA* regional citrate anticoagulation, *RRT* renal replacement therapies, *CRRT* continuous renal replacement therapies

Considering that circuit anticoagulation with citrate is based on calcium chelation in the blood of the extracorporeal circulation and that a proportion of calcium-citrate complexes are lost in the effluent, it is well known that all RCA-RRT modalities are characterized by a negative mass balance of calcium [26]. Hence, an appropriate calcium supplementation is needed to avoid dangerous systemic hypocalcemia and calcium depletion during RCA. According to different RCA-CRRT protocols and parameter settings (e.g., citrate dose, effluent flow rate), systemic calcium supplementation needs may vary considerably, ranging from 3 up to 5 mmol/h with the use of specifically dedicated calcium-free solutions. Calcium supplementation can be infused in a separate central venous line or in a circuit-integrated post-filter line; its infusion rate is titrated to maintain systemic ionized calcium in the normal range (1.1–1.25 mmol/l). Both 10% calcium chloride or 10% calcium gluconate solutions can be used considering that these solutions provide different amounts of elemental calcium (0.68 and 0.226 mmol/ml, respectively) [26].

In this regard, appropriately designed RCA protocols could allow to minimize the need for calcium supplementation by coupling the use of citrate solutions with calcium-containing dialysate and/or replacement fluids. This strategy may consistently reduce the amount of calcium supplementation and could delay the occurrence of hypocalcemia in case of unintentional interruption of calcium infusion. This approach, although carrying the need to accept a higher target of ionized calcium in the circuit, still appears compatible with a prolonged filter lifespan [14, 56, 84–86]; in any case, additional checking of post-filter calcium concentration may be useful to assess the need for adjusting parameter settings (dialysate flow rate, citrate dose) to meet the intended target (< 0.50 mmol/l).

Because phosphorus is not a standard component of CRRT fluids, hypophosphatemia represents a common electrolyte derangement among critically ill patients undergoing CRRT or PIRRT [43, 87–89]. Considering the unfavourable clinical impact of phosphate depletion, any measure aimed at reducing the incidence and severity of RRT-related hypophosphatemia should be implemented. In this regard, especially in patients undergoing prolonged RRT modalities, it seems appropriate to prevent the occurrence of hypophosphatemia through the use of phosphate-containing CRRT solutions [88, 89]. Phosphate-containing solutions are now available in many Countries and their adoption has been reported as safe and effective in preventing CRRT-induced hypophosphatemia [90] also in the setting of RCA [84]. In the daily practice, the proportion of dialysis dose given as phosphate-containing solution should be tailored to the single patient and to the evolving clinical setting. The use of phosphate-containing dialysate and/or replacement fluids as a high proportion of dialysis dose may be associated with mild hyperphosphatemia in some patients [91]. In the specific setting of RCA, this issue could potentially occur in protocols characterized by the use of high concentration citrate solutions if the whole CRRT dose is reached by using a phosphate-containing solution as the sole CRRT fluid. Conversely, the risk of hyperphosphatemia is negligible with RCA protocols based on the adoption of low-concentration citrate solutions, in which phosphate-free pre-dilution citrate flow rate accounts for roughly 50% of dialysis dose [55].

In commercially available CRRT fluids, magnesium concentration ranges from 0.5 to 0.75 mmol/l and, in conventional CRRT modalities, this concentration generally allows to maintain serum magnesemia within normal ranges [92]. However, this statement does not apply to RCA, which include hypomagnesemia among its potential complications [26]. Indeed, as reported for ionized calcium, also ionized magnesium is chelated by citrate and is partially removed with the effluent fluid, generating a negative magnesium balance which may vary in relation to CRRT dose, RCA protocol adopted (low- or high-concentration citrate solutions) and magnesium concentration in the dialysate/replacement fluid [26, 92]. In this regard, since magnesium loss in RCA-CVVHDF is only partially balanced by the concentration of magnesium in commercially available dialysis/substitution fluids, RCA may lead to the depletion of magnesium body pool [93]. This issue, more frequently occurring in protocols adopting low concentration citrate solutions (delivery of a consistent proportion of CRRT dose as magnesium-free citrate solution), may be prevented by including parenteral magnesium sulphate supplementation in the routine procedures of RCA protocol [84]. In the setting of an RCA-CVVHD protocol adopting a high concentration citrate solution, it has been shown that the use of a 0.75 mmol/l magnesium concentration in the dialysate avoided the need for magnesium supplementation; however, a slightly negative magnesium balance may occur, leading to a stabilization of serum magnesium concentration in the low normal range [52]. Taking into account these findings, a further optimization of magnesium concentrations in CRRT fluids should be included among the targets of future developments of RCA-CRRT protocols. In summary, the choice of different combinations of nowadays-available CRRT solutions allows to tailor CRRT prescription to patient’s needs and to significantly reduce the risk of electrolyte derangements. However, further refinements of the composition of CRRT and citrate solutions could help to minimize calcium, magnesium, and phosphate supplementations, with the aim to reduce the need for additional interventions, thus simplifying the management of the RCA-CRRT.

**Table:** 

Summary of expert panel opinion
Section 1
Should regional citrate anticoagulation (RCA) be considered as the first-choice anticoagulation strategy during Continuous Renal Replacement Therapies (CRRT)? If allowed by local resources, based on current evidence, RCA should be considered as the first-choice anticoagulation strategy, provided that limitations to the use of citrate have been carefully evaluated (see “Section 4. Potential limitations to RCA and monitoring in high-risk patients” section)
Section 2
Can be RCA protocols be applied to all different RRT modalities? With properly designed protocols, RCA can be safely applied in all RRT modalities, including PIRRT Low- or high-concentration citrate solutions can be indifferently selected for all RRT modalities? - High-concentration citrate solutions can be potentially used in all RRT modalities (diffusive, convective, and mixed) but are preferentially used in the diffusive ones - Low-concentration citrate solutions represent part of dialysis dose (as pre-dilution replacement fluid) and are consequently compatible only with RCA protocols designed for convective or mixed RRT modalities
Section 3
Is RCA applicable in specific extracorporeal organ support systems combined with RRT? - The use of RCA for anticoagulation of integrated systems including extracorporeal CO2 removal (ECCO2R) and CRRT requires further studies aimed at designing specific protocols. The citrate load related to the high blood flow which characterizes ECCO2R appears the main limit for its application in this setting - The use of RCA for anticoagulation of albumin dialysis circuit has been successfully reported in patients with liver failure. However, the risk of citrate accumulation requires a careful monitoring Is RCA applicable in course of extracorporeal membrane oxygenation (ECMO)? The use of RCA for the anticoagulation of CRRT circuit in the setting of ECMO (integrated or handled in a parallel system by using a separate vascular access) is feasible and allows a prolonged filter lifespan
Section 4
Do relative or absolute contraindications to RCA exist? RCA should be avoided in patients with worsening lactic acidosis regardless of its cause (e.g., inadequate oxygen delivery, impaired liver clearance, mitochondrial dysfunction) During RCA, which parameters should be monitored to identify citrate accumulation/toxicity? Systemic ionized calcium (iCa) and acid–base parameters must be routinely measured to assess a trend towards hypocalcemia and worsening metabolic acidosis; total Ca/iCa ratio should be promptly calculated to confirm citrate accumulation (threshold > 2.5) How can the conditions of citrate net overload and insufficient citrate load be distinguished from citrate accumulation/toxicity? Differently to citrate accumulation/toxicity—characterized by the triad systemic ionized hypocalcemia, increasing need for calcium supplementation, and worsening metabolic acidosis—citrate net overload and insufficient citrate load represent easily manageable conditions related to acid–base imbalances due to suboptimal RCA-CRRT parameters setting (see Table 2 for details and corrective interventions)
Section 5
Can calcium-containing solutions be used in specifically designed RCA protocols? Specifically designed RCA protocols, including calcium-containing dialysate and/or replacement fluids, allow to minimize the need for calcium supplementation and appear still able to ensure a prolonged filter lifespan Can phosphate-containing solutions be used in the setting of RCA-CRRT? The adoption of phosphate-containing solutions as a quote of dialysis dose has been reported as safe and effective in preventing CRRT-induced hypophosphatemia also in the setting of RCA Could an even magnesium balance be obtained with available CRRT solutions when properly designed RCA-CRRT protocols are applied? The loss of magnesium during RCA is only partially balanced by the standard commercially available CRRT solutions; further refinements of magnesium concentration in RCA-dedicated CRRT fluids are needed to approach an even magnesium balance, especially in protocols adopting low concentration citrate solutions
